# Differences in influenza testing and treatment in micropolitan versus metropolitan areas in the U.S. using medicare claims data from 2010 to 2016

**DOI:** 10.1186/s12889-025-21555-4

**Published:** 2025-01-23

**Authors:** Alexia Couture, F. Scott Dahlgren, Hector S. Izurieta, Richard A. Forshee, Yun Lu, Carrie Reed

**Affiliations:** 1 Epidemiology and Prevention Branch, Influenza Division, National Center for Immunization and Respiratory Diseases, Centers for Disease Control and Prevention, 1600 Clifton Rd, MS 24/7, Atlanta, GA 30329-4027 USA; 2 Faculty of Electrical Engineering, Mathematics and Computer Science, Delft University of Technology, Delft, The Netherlands; 3 Center for Biologics Evaluation and Research, Food and Drug Administration, Silver Spring, MD USA

**Keywords:** Influenza, Antivirals, Rapid-testing, Rurality

## Abstract

**Background:**

To improve understanding of influenza and rurality, we investigated differences in influenza testing and anti-viral treatment rates between micropolitan (muSAs) and metropolitan statistical areas (MSAs) using national medical claims data over multiple influenza seasons.

**Methods:**

Using billing data from the Centers for Medicare and Medicaid Services for those aged 65 years and older, we estimated weekly rates of ordered rapid influenza diagnostic tests (RIDT) and antivirals (AV) among Medicare enrollees by core-based statistical areas (CBSAs) during 2010–2016. We used Negative Binomial generalized mixed models to estimate adjusted rate ratios (aRR) between MSAs and muSAs, adjusting for clustering by CBSA plus explanatory variables. We ran models for all weeks and only high influenza activity weeks.

**Results:**

For all weeks, the unadjusted rate of RIDTs was 1.97 per 10,000 people in MSAs compared with 2.69 in muSAs (Rate ratio (RR) = 0.73, 95% Confidence Interval (CI): 0.73–0.74) and of AVs was 1.85 in MSAs compared with 1.40 in muSAs (RR = 1.32, CI: 1.31–1.32). From the multivariate model, aRR for RIDTs was 0.82 (0.73–0.94) and for AVs was 1.12 (1.04–1.22) in MSAs versus muSAs. For high influenza activity weeks, aRR for RIDTs was 0.82 (0.73–0.92) and for AVs was 1.15 (1.06–1.24). All models found influenza testing rates higher in muSAs and treatment rates higher in MSAs.

**Conclusions:**

Our study found lower testing and higher treatment in U.S. metropolitan versus micropolitan areas from 2010 to 2016 for those aged 65 years and older in our population. Identifying differences in influenza rates by rurality may improve public health response. Further research into the relationship of rurality and health disparities is needed.

**Supplementary Information:**

The online version contains supplementary material available at 10.1186/s12889-025-21555-4.

## Background

Between 2010 and 2023, influenza-related illnesses per year in the U.S. ranged from an estimated 9.3 to 41 million, with estimated hospitalizations ranging from 100,000 to 710,000 [1]. Influenza outbreaks are influenced by multiple variables, including viral evolution, genetics, behavior changes associated with influenza and other epidemics and pandemics, geography, and socioeconomic factors [2–6]. Impact of influenza based on geography has been studied mostly through either large populations, usually utilizing surveillance data captured in urban or suburban areas, or through site specific, finite populations at single state/local levels [2, 3, 7–10]. Understanding differences of multiple influenza indicators specifically in rural versus urban cores could inform public health response and resource allocation for influenza.

Differences in healthcare between rural and urban areas in the U.S. are evident from many studies with ranging topics, including the prevalence of chronic conditions, risky health behaviors, reproductive health, mortality, and vaccination rates [11–21]. The many studies reporting differences have discussed both access and behavior as possible explanations for discrepancies [11–21]. Population beliefs regarding care-seeking, vaccination, and disease severity may also contribute to the differences. However, influenza outbreaks in rural areas remain largely unstudied, especially looking at testing and treatment of influenza. Rural areas have gaps in healthcare delivery which may be exacerbated by or influential on influenza outbreaks, especially when compared to urban areas [22]. However, rural areas may also be protected from large outbreaks given lower population density since influenza progression is highly based on human mobility [23]. Few studies have focused on quantitatively analyzing how influenza differs by population size and rurality. One study utilized Shannon’s entropy to describe outbreak intensity [24]. Dalziel and colleagues calculated Shannon’s entropy to compare differences in influenza outbreak intensity between small and large cites. A study focused in Colorado used census tract data to examine influenza rates in rural versus urban areas [25]. Also, a Missouri study examined rurality and differences in sociodemographic factors for the 1918-20 influenza pandemic [7]. Very few other studies have compared influenza outbreaks between micropolitan (< 10,000 residents) versus metropolitan (≥ 10,000 residents) areas. Further, there are also very few sources of nationwide data that is representative of rural areas on trends in the use of influenza testing or influenza antivirals and thus limited understanding of how their use varies by rurality. Investigating differences in influenza trends by population size and rurality and across multiple indicators adds to a needed and understudied area of public health. Over the years, enormous progress to electronic disease surveillance systems and technologies has allowed for collection of more data and information on the epidemiology of influenza than ever before.

For our study, we used national medical claims data of rapid influenza diagnostic test (RIDT) and oseltamivir prescriptions, an antiviral (AV) drug used to treat influenza, dispensed to Medicare Part D enrollees and compared influenza testing and treatment rates between metropolitan versus micropolitan areas of the U.S. Past analysis found that the use of oseltamivir dispensing in medical claims data trends closely with other indicators of influenza activity, like the proportion of influenza-like illnesses among outpatient visits in the US [26]. However, it has also been shown that the ordering of RIDTs versus AVs vary by medical specialty; for example, the odds of a physician ordering an RIDT were higher than ordering an AV prescription in ER or pediatric settings [27, 28]. Looking at both testing and treatment may offer a more compressive view of differences in influenza by rurality. Using data from across the U.S. and over multiple influenza seasons, we investigate the differences in rates of influenza testing and treatment between micropolitan and metropolitan areas while controlling for other influential factors on influenza outbreaks, including weather, population, sociodemographics, and seasonality.

## Methods

### Core-based statistical areas

The U.S. Census Bureau and the Office of Management and Budget defined core based statistical areas (CBSAs) as groups of counties with a common urban core with at least 10,000 residents [29, 30]. CBSAs are categorized as Metropolitan statistical areas (MSAs) or Micropolitan statistical areas (muSAs). Metropolitan statistical areas (MSAs) have an urban core of 50,000 residents or more. Micropolitan statistical areas (muSAs) have an urban core of at least 10,000 but less than 50,000 residents. The definitions also included adjacent counties with strong economic or social ties into these CBASs. Our paper used the rural definition by the Office of Management and Budget: all nonmetropolitan areas are considered rural, i.e. muSAs are considered rural [29]. According to the July 15, 2015, definitions, there are 945 CBSAs, which about 96% of the U.S. population lived in during 2010–2016. The rest of the population live in counties that do not meet the CBSA defined criteria. Of the 945 CBSAs, there were 556 muSAs and 389 MSAs.

### Testing and treatment data

The Centers for Medicare and Medicaid Services (CMS) pay for medical care delivered to millions of people in the United States. Using billing data from CMS of those 65 years and older, data from outpatient visit claims on ordered RIDTs and prescriptions of oseltamivir AVs dispensed for treating influenza among beneficiaries were extracted. Weekly numbers of RIDTs and AVs dispensed to beneficiaries are tabulated by CBSA from October 3, 2010, to August 6, 2016, influenza seasons 2010–2011 through 2015–2016. The same data has been used in other studies as a proxy for influenza with further explanation of data available [26, 31]. 

### Prescription drug plan data

CMS tabulates the number of people enrolled in Prescription Drug Plans (PDP) in each county and publishes these data each month. We linearly interpolated the monthly estimates to obtain weekly estimates. We aggregated the county level estimates to the CBSA level. We considered the estimated number of enrollees each week as the population at risk, and we used these weekly estimates as an offset in our model to estimate rates of dispensed RIDTs and AVs. Having the population offset change overtime offered a more real-time, dynamic estimate. We also considered a static offset of the population aged 65 years and older in the CBSA but found the offset of enrollees that we used is more likely the correct denominator for the numerator to get calculated rates.

### Weather data

The National Oceanic and Atmospheric Administration publishes the Global Surface Summary of the Day, which contains weather data from over 9,000 stations around the world. These data include temperature and dew point, which we used to compute absolute humidity. Previous research demonstrated low temperature and low absolute humidity increase the airborne transmission of influenza [5]. We only considered data from stations which are largely (> 90%) complete for these two variables. We lagged these data by 5 days to account for the time from infection to the time to dispensing antivirals, which includes 3 day average of symptom onset from infection and 2 day recommended AV treatment after symptom onset. Then, we interpolated any missing daily values and averaged the daily values to construct a time series of weekly values. We included these weekly temperature and weekly absolute humidity for variable selection when building our model of the weekly rate of antivirals dispensed to beneficiaries. If no stations were in the CBSA, we chose the closest one. If more than one station was in the CBSA, we averaged values from all stations in the CBSA for the final temperature and humidity variables.

### Census data

The United States Census Bureau publishes population estimates each year by county and geometry by CBSAs [30, 32]. We added these population estimates to obtain estimates of the total population within CBSAs. We calculated area (meters squared) from geometry for each CBSA. Then, we calculated population density from population and area in each CBSA.

The U.S. Census Bureau also has the American Community Survey that releases demographic data by CBSA. We used data from the 2012 and 2017 5-year surveys. Specifically, we used yearly population density, percent of population over 85 years, percent of people whose income fell below the poverty line, percent of population aged 16 + that is unemployed, percent of population aged 25 + with high school education, percent of population aged 65 + with only Medicare, percent of population that own their home, percent of population with no vehicle, percent of people that have a 60 + minute commute to work, median house value, and percent of population that is non-white to control for differences between CBSAs when building our model.

### Influenza activity data

The Centers for Disease Control and Prevention (CDC) publishes data from the U.S. Outpatient Influenza-like Illness Surveillance Network (ILINet) [33, 34]. ILINet is a nationwide sentinel surveillance system from health care providers that reports weekly outpatient visits for influenza-like illness (ILI). ILINet data were used to produce a weekly level of ILI activity for each jurisdiction, ranging from Minimal to High Activity [33, 34]. For our secondary analysis, we matched those defined levels of ILI activity to our weekly CMS data by week and state and then filtered weeks in our data to only be High ILI Activity weeks to aid in understanding differences of influenza.

### Statistical modelling

Unadjusted weekly rates of RIDTs and AVs dispensed to beneficiaries were calculated for MSAs and muSAs. To better handle over-dispersion from our data, we ran Negative Binomial generalized mixed models. First, we ran a Negative Binomial model to estimate rates. Second, we ran Negative Binomial generalized mixed models with a random intercept to account for clustering in the data to calculate adjusted rates. We tried a random intercept for CBSAs to account for correlation of RIDTS or AVs within CBSAs. Third, we ran Negative Binomial spatial models for spatially smoothed adjusted rates, specifically the Besag-York-Mollie model that uses the conditional autoregressive (CAR) distribution [35]. Models were built for each outcome, the weekly number of ordered RIDTs and prescribed AVs. The predictor variable was the binary variable of MSA versus muSA to get relative rates. The model included an offset of the population enrolled in a PDP in each CBSA so the outcomes of the model were rates per 10,000 people enrolled in a PDP. Fourth, we introduced explanatory variables to account for demographic and temporality and ran multivariate Negative Binomial generalized models with whichever model above had the best fit based on the Widely Applicable Information Criterion (WAIC) [36]. 

We considered the following centered and standardized variables for selection:

- Temperature.
- Absolute humidity.
- Population density within the CBSA.
- Percent population over 85 years old.
- Percent of adult population that fell below poverty threshold in 2016.
- Percent of population aged 16 + that is unemployed.
- Percent of population aged 25 + with high school education.
- Percent of population aged 65 + with only Medicare.
- Percent of population that own their home.
- Percent of population with no vehicle.
- Percent of people that have a 60 + minute commute to work.
- Median house value.
- Percent of population that is non-white.

We also considered season variables which accounts for the periodic nature of influenza seasons anddifferences between influenza seasons:

- Influenza season.
- Fourier-Serfling terms: cos (2πt/52.25), sin (2πt/52.25), cos (4πt/52.25), and sin (4πt/52.25).
- Interaction terms for the effects of season and the Fourier-Serfling terms [37].

We ran models for both RIDTs and AVs on all weeks. As a secondary analysis, we ran models for both RIDTs and AVs for only weeks with High ILI Activity. For those models, we did not include the Fourier Serfling terms because cyclical/seasonal data trends were removed. Correlation analysis was run on all covariates to check for multi-collinearity.

Finally, we computed Shannon’s entropy to compare with previous literature [24], for each season and each CBSA. Shannon defined entropy as an axiomatic measure of how much information a discrete process produces [38]. In the context of this paper, one may refine a seasonal number of influenza cases into monthly cases, into weekly cases, into daily cases; until eventually, we have a partition where every interval of time has at most one case. Weekly time series of influenza has more information than monthly time series. For a given CBSA during a season with *W* weeks, if $\:n\:=\:{n}_{1}\:+\:\dots\:\:+\:{n}_{W}$ are the weekly antivirals, then the Shannon entropy is $\:{\sum\:}_{w=1}^{W\:}\:\frac{{\:n}_{w}}{nlog\left(\frac{n}{{\:n}_{w}}\right)}\:\:\:$. Dalziel et al. defined epidemic intensity as the inverse of this value, which would be lower in areas where incidence is distributed evenly across weeks and higher when more of an area’s incidence is more concentrated in fewer weeks [24]. 

We used R version 4.1.2 and 4.2.2 for all computations. For the models, we used integrated nested Laplace Approximation using the R-INLA package. For more information on assumptions and default priors for INLA models, please refer to the R-INLA reference [39]. In order to compare model fit, we calculate and compare the WAIC. The lower the value of WAIC, the better the model fit.

## Results

**Fig. 1:**
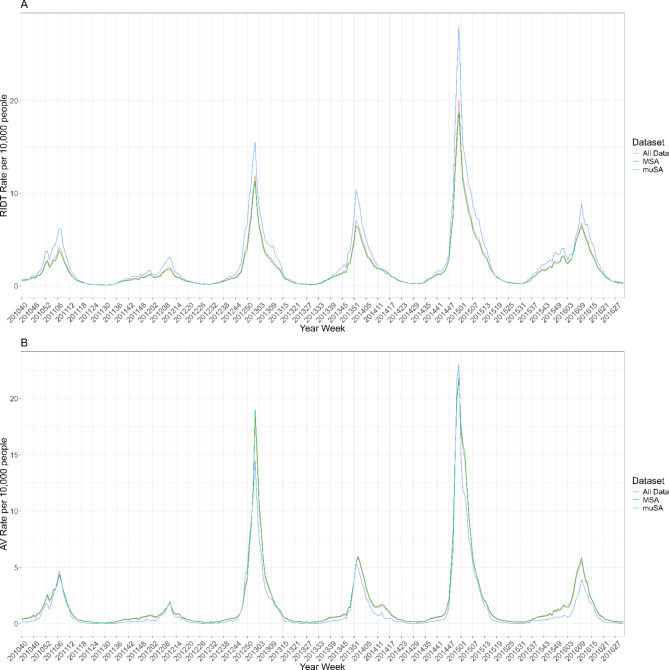
Weekly rates of (**A**) Rapid Influenza Diagnostic Tests (RIDTs) and (**B**) Anti-Virals (AVs) per 10,000 people for all CBSAs combined and stratified into metropolitan statistical areas (MSAs) and micropolitan statistical areas (muSAs)

Our final dataset consisted of 288,225 weekly observations of CMS data from 945 CBSAs. Of the 945 CBSAs, 389 were MSAs and 556 were muSAs. Figure 1 shows the weekly rates, over time, of the dispensing of RIDTs and AVs for all CBSAs and by MSA/muSA for those 65 + years. In the secondary analyses, high ILI activity weeks consisted of 17,789 weekly observations from 927 CBSAs from the U.S.; 18 were excluded from this analysis due to not having high ILI activity in any of the included seasons. Of the 927 CBSAs, 384 were MSAs and 543 were muSAs.

Among enrollees in a PDP, 65 + years of age living within a CBSA, the unadjusted rate of influenza testing with RIDTs for all weeks was 2.07 per 10,000 people per week, and the unadjusted rate of AV use was 1.78 per 10,000 people. During only High ILI activity weeks, the unadjusted rate of testing with RIDTs was 10.87 per 10,000 people per week, and the unadjusted rate of AV use was 11.02 per 10,000 people.

When stratifying by the size of the urban core of the CBSA, for all weeks, the unadjusted rate of RIDTs was 1.97 in MSAs compared with 2.69 in muSAs (Rate Ratio (RR) = 0.73, 95% Confidence Interval (CI): 0.73, 0.74). For all weeks, the unadjusted rate of AV use was 1.85 per 10,000 people in MSAs compared with 1.40 per 10,000 people in muSAs (RR = 1.32, 95% CI: 1.31, 1.32).

**Fig. 2:**
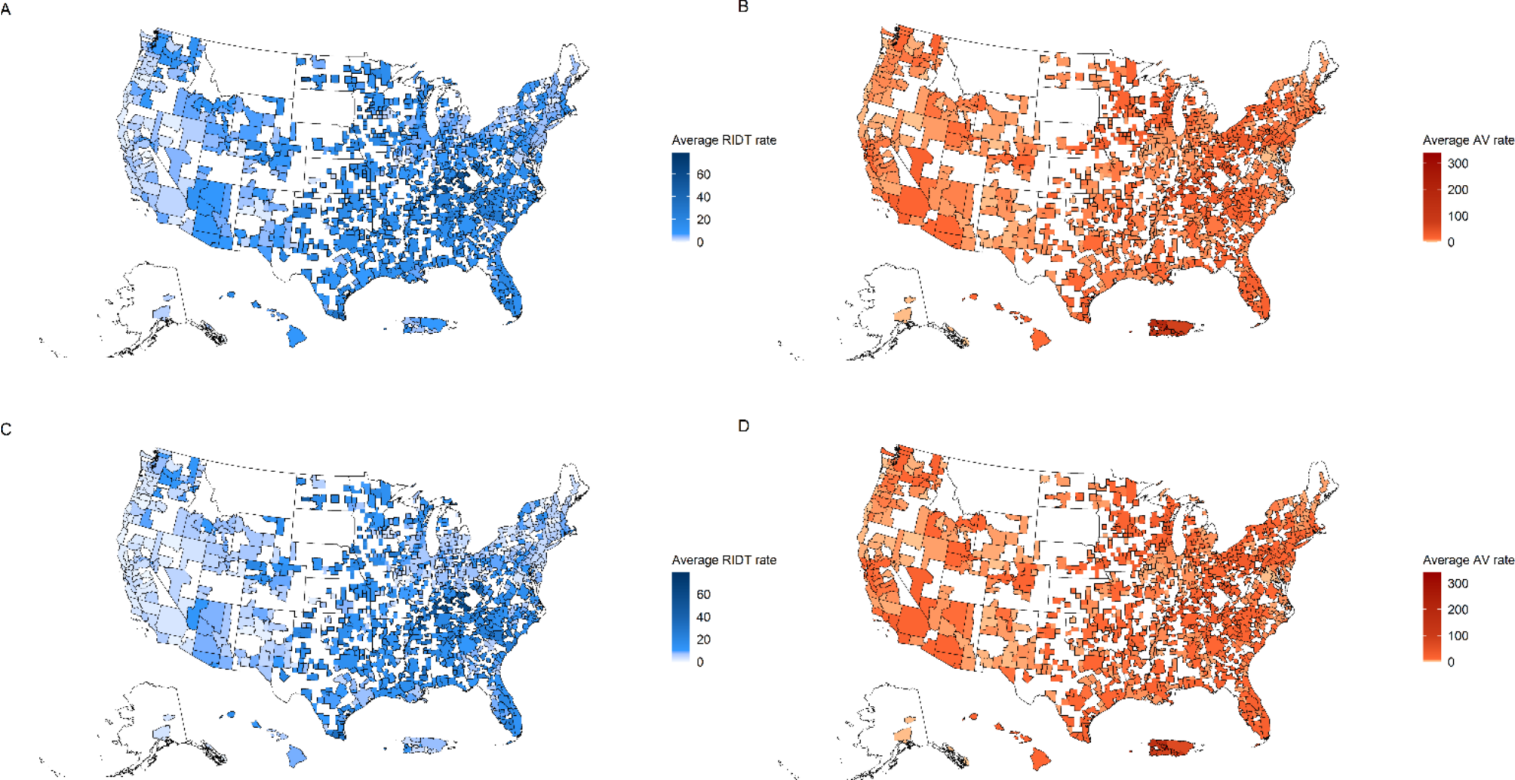
Heatmap of Core Based Statistical Areas’ (CBSA’s) weekly rates of (**A**) Rapid Influenza Diagnostic Tests (RIDTs) and (**B**) Anti-Virals (Avs) per 10,000 people for all weeks combined and (**C**) RIDTs and (**D**) AVs per 10,000 people for only High Influenza-like-illness (ILI) weeks. Areas in white do not belong to a CBSA or were excluded for only High ILI weeks

When only considering high ILI weeks, the unadjusted rate of RIDTs was 10.23 per 10,000 people in MSAs compared with 14.19 per 10,000 people in muSAs (RR = 0.72; 95% CI: 0.72, 0.73). For only High ILI weeks, the unadjusted rate of AV use was 11.27 per 10,000 people in MSAs compared with 9.74 per 10,000 in muSAs (RR = 1.16; 95% CI: 1.15, 1.17). Figure 2 shows a map of each CBSA’s overall weekly rates of dispensing RIDTs and AVs for all weeks and only High ILI weeks. Supplemental Fig. 1 shows which CBSAs are muSAs versus MSAs.

**Fig. 3:**
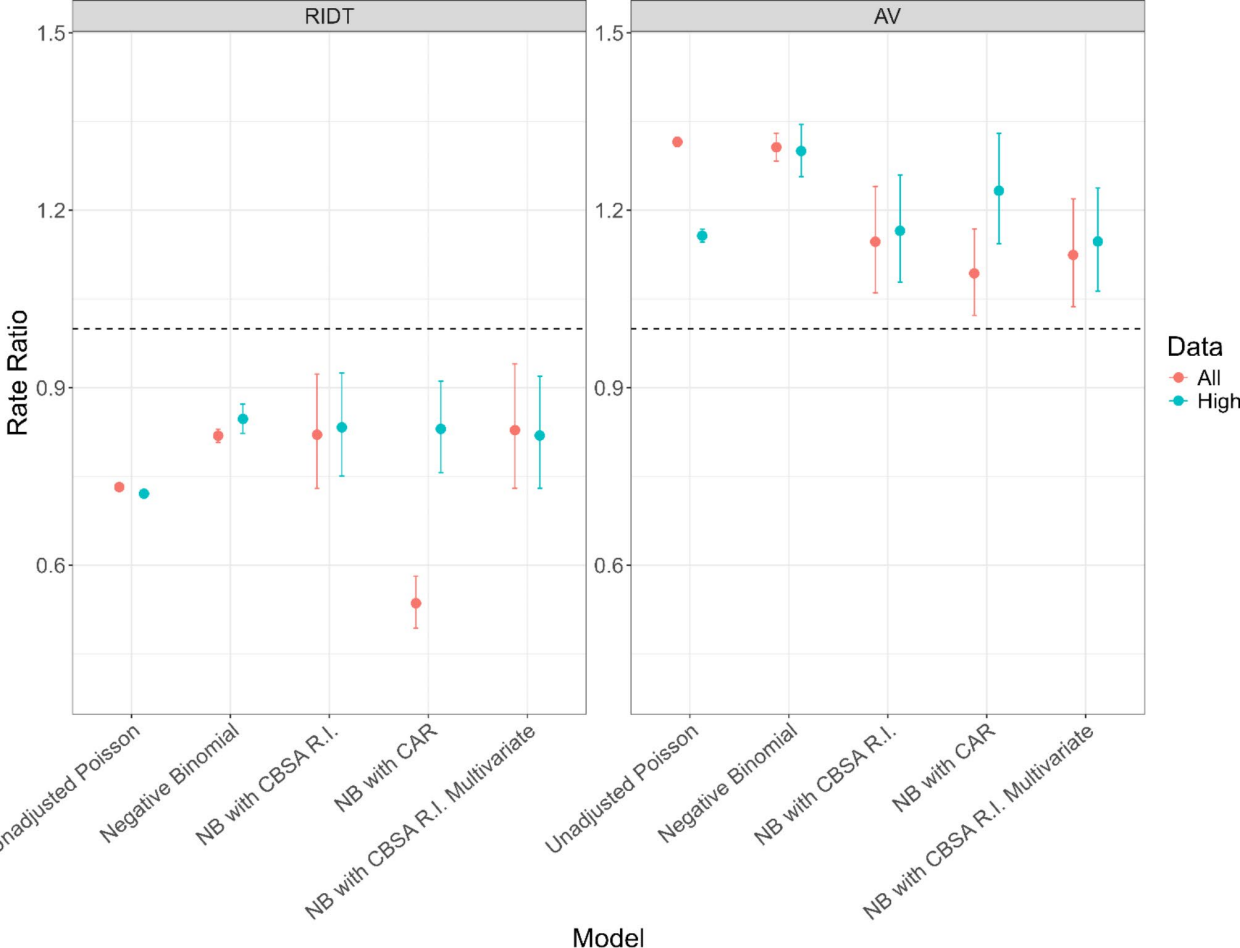
Rate ratios or adjusted rate ratios and 95% confidence intervals of dispensing Rapid Influenza Diagnostic Tests (RIDTs) and Anti-Virals (Avs) weekly in metropolitan statistical areas (MSA) micropolitan statistical areas (muSAs) for all weeks (red) and only High Influenza-like-illness (ILI) weeks (blue) from unadjusted calculation, negative binomial models with random intercepts, spatial smoothing using Conditional Autoregressive (CAR) distribution, and multivariate negative binomial model

We considered various modeling strategies, and while there were some variations in the point estimates across models (ranging from 0.54 to 0.85 for RIDTs and 1.09–1.32 for AVs), the trends and directionality of the associations remained consistent. See Table 1; Fig. 3 for rate ratios from all analysis. When looking at correlation of covariates, we found all correlations to be extremely low and statistically insignificant, except for temperature and humidity. Since environmental evidence shows both temperature and humidity to be important to influenza epidemics, we left both in the models. For all data and only high ILI weeks, the model with covariates and random intercept CBSAs had the lowest WAIC for both RIDT and AV (Table 1). Multivariate models were built with CBSA random intercepts since they had the lowest WAIC of the models of the bivariate models. Full multivariate coefficients can be found in the Supplemental Table 1.

**Table 1:** Rate ratio of weekly rates of dispensing Rapid Influenza Diagnostic Test (RIDT) and anti-virals (AV) in metropolitan statistical areas (MSA) versus micropolitan statistical areas (muSAs), 95% confidence intervals, and widely Applicable Information Criteria from multiple models

		All Weeks		High ILI Activity Only	
Outcome	Model	Rate Ratio (95% CI)	WAIC	Rate Ratio (95% CI)	WAIC
RIDT	Unadjusted Poisson	0.73 (0.73–0.74)	2,212,847	0.72 (0.72–0.73)	291,711
	Negative Binomial	0.82 (0.81–0.83)	1,079,306	0.85 (0.82–0.87)	117,834
	NB with CBSA intercept	0.82 (0.73–0.92)	1,018,566	0.83 (0.75–0.92)	111,114
	NB with spatial CAR	0.53 (0.49–0.58)	1,020,019	0.83 (0.76–0.91)	111,237
	Multivariate NB with CBSA intercept	0.83 (0.73–0.94)	805,894	0.82 (0.73–0.92)	103,493
AV	Unadjusted Poisson	1.32 (1.31–1.32)	35,782,690	1.16 (1.15–1.17)	1,003,286
	Negative Binomial	1.31 (1.28–1.33)	840,838	1.30 (1.26–1.35)	113,818
	NB with CBSA intercept	1.15 (1.06–1.24)	820,023	1.16 (1.07–1.25)	109,506
	NB with spatial CAR	1.09 (1.02–1.17)	820,182	1.23 (1.14–1.33)	109,626
	Multivariate NB with CBSA intercept	1.12 (1.04–1.22)	641,016	1.15 (1.06–1.24)	103,322

Footnote: NB – Negative Binomial, CI – confidence interval, WAIC – Widely Applicable Information Criteria, CAR – Conditional Autoregressive

Finally, we calculated the average Shannon entropy. We found the average Shannon entropy among MSAs was greater than muSAs each season for both RIDTs and AVs for all weeks (Table 2), although there were some differences in the average Shannon entropy each season depending on which indicator was used. The results showed an increase in entropy across seasons as well.

**Table 2:** Average Shannon Entropy comparison between metropolitan statistical areas (MSAs) and micropolitan statistical areas (muSAs) for each influenza season and corresponding *p*-value

Season	Ordered Rapid Influenza Diagnostic Tests			Prescribed Antivirals		
	MSAs	muSAs	*p*-value	MSAs	muSAs	*p*-value
2010–2011	2.41	1.67	e^− 84^	2.55	1.71	e^− 163^
2011–2012	2.63	1.78	e^− 94^	2.52	1.30	e^− 239^
2012–2013	2.58	2.05	e^− 64^	2.66	2.26	e^− 108^
2013–2014	2.82	2.15	e^− 94^	2.84	2.07	e^− 189^
2014–2015	2.67	2.30	e^− 54^	2.73	2.40	e^− 114^
2015–2016	2.95	2.50	e^− 66^	2.91	2.13	e^− 212^

## Discussion

Using nationwide community setting claims from Medicare enrollees aged 65 years and older, we consistently found the weekly rate of influenza testing with RIDTs was higher in micropolitan areas with a small urban core relative to metropolitan areas with a large urban core; however, we also saw the opposite relationship with the weekly rate of dispensing antiviral medications for treating influenza, which was higher in MSAs relative to muSAs. This was true for all weeks in the analysis as well as when limiting to those weeks with high influenza-like-illness activity. After controlling for factors available to us that may be drivers of influenza activity including meteorological, sociodemographic, and across season variability, the adjusted estimate of the rate ratio of MSA to muSA for both indicators remained consistent in directional trends with an extremely small magnitude change. These results give consistent evidence that, among Medicare enrollees, there was variation in influenza testing and treatment, with influenza testing with rapid antigen tests higher in small urban core areas while rates of influenza treatment with oseltamivir were higher in large urban core areas. Acknowledging that numerous factors influence testing and treatment that we were not able to include as explanatory variables due to unavailable data, we limit our discussion to our finding of differences and offer potential explanations that need further mixed methods analysis to fully understand.

The rate ratio for treatment trends with how influenza has been described to spread through large and dense populations [2, 3, 8–10]. Since we do not have testing outcome data, the higher treatment rates could be due to higher positive rates in urban areas. They could also be due to a difference in approach between urban clinicians and rural clinicians. However, the finding of higher testing rates in rural areas stand regardless of positive or negative outcomes, perhaps highlighting a discrepancy in urban use of RIDTs. Although limited as a comparison, research on COVID-19 has also reported lower rates and access of Paxlovid in rural areas [40]. Finding testing to be higher in small urban areas while treatment to be higher in large urban areas allows for the possibility of influenza intensity being high in either/both places, but nonetheless, it highlights the difference that exists between rural and urban areas. This evidence supports both the quantitative and qualitative need for further investigation on multiple influenza indicators and health behaviors.

Understanding why we see differences in influenza testing and treatment when examining rurality may be due to many complex logistical and behavioral factors. Despite finding rates of influenza testing to be higher in micropolitan compared to metropolitan areas, rates of antiviral usage were lower. One potential explanation for this discrepancy could be that urban areas are more selective with testing and produce higher positive rates therefore needing more treatment. A previous study defined a positive case as receiving testing and treatment, which highlights the potential explanation of higher treatment meaning higher positives [41]. However, another potential explanation could be that rural areas have delayed care-seeking and therefore receive treatment less than urban areas. These are speculations, and many more scenarios could be proposed. Access to a physician, influenza diagnostic testing, prescription drugs, enrollment in a PDP, and rurality are entangled in a complex way [42]. Recent research on the gaps in rural community healthcare access found higher proportion of higher health risk populations, including populations age 65 and older, in micropolitan areas without pharmacies [43]. People may elect to enroll in a PDP to receive prescription drugs through the mail or by courier when there are no nearby pharmacies. However, even people with partial access to prescription drugs may not be able to fill a prescription for antivirals within two days of their onset of illness. Furthermore, potential distrust or other behavioral barriers may also contribute to the lower treatment rates in rural areas compared to the testing rates. Dispensing of COVID-19 antivirals was reported to be lower in socially vulnerable communities even when they had many dispensing sites [44]. To truly answer why there is this discrepancy in testing and treatment lies beyond the scope of our analysis. However, by looking at both testing and treatment rates for influenza together, rather than just a single indicator of influenza burden or intensity we gain greater insights into potential discrepancies and resource limitations by rurality.

A previous report by Dalziel and colleagues found greater Shannon entropy in the time series of influenza from larger cities relative to smaller cities [24]. We likewise found a similar pattern with greater Shannon entropy in MSAs relative to muSAs for both RIDTs and AVs. They interpret this finding to mean larger cities have more diffuse epidemics of influenza and that influenza activity is concentrated in fewer weeks in rural areas compared with urban areas. However, we attribute the pattern in entropy to combinatorics instead of a significant observation about the epidemiology of influenza. When the population at risk is larger, the number of RIDTs and AVs dispensed each season tends to be higher; the number of ways to refine the seasonal counts into weekly counts tends to be higher; and the Shannon entropy tends to be higher. If the number of seasonal RIDTs or AVs dispensed in every CBSA had been much larger, then applying the interpretation of Dalziel and colleagues to our results may have been appropriate. However, we take a basic interpretation of the Shannon entropy: data on influenza from larger populations contains more information than data about smaller populations. Since using this calculation to represent influenza intensity is not informative, we wanted to focus on understanding influenza discrepancies by rurality and analyzed RIDTs and AVs as rate ratios to compare between MSAs and muSAs.

Since our study uses RIDTs and AVs from CMS data as indicators for influenza, we are limited in generalizability by access to healthcare, prescription drugs, and Medicare population. First, our findings are from the Medicare population so differences found for treatment and testing, although adjusted for multiple variables, are limited in generalizability to the entire U.S. population. Within this limitation, our analysis of only the population 65 + years of age cannot be generalized to all ages. Second, even within our data, although the proportion of people enrolled in a Medicare PDP among those eligible to enroll increased from 2009 to 2017, it varied widely across states and was lower among rural counties [45]. Although we controlled for the changing number of people enrolled in a PDP in our analysis, some confounding by this access to care in rural areas must remain. Third, we do not have access to test results so are limited in interpretation of testing and treatment rates and cannot compare it to positivity rates. Fourth, although we adjusted for meteorological, sociodemographic, and seasonality variables, we acknowledge that there may be important factors not incorporated in the models, including vaccination data which were not available at the CBSA level. We want to note that HHS region or other geographical differences may have an impact that is not considered in this analysis. Along with this, the variables we do have, specifically from the ACS, may also have limitations since they are small area estimations and we only have them from 2012 to 2017 since it is a 5 year survey. Fifth, we only had data on oseltamivir prescriptions, so we do not know the trends of other AV prescriptions or home remedies and how they differ between MSAs and muSAs. Sixth, since we linearly interpolated monthly estimates to obtain weekly estimates, we may have masked fluctuations within a month. We also interpolated missing weather daily values, which may not capture random daily fluctuations. Finally, although we spanned multiple seasons, our study used data prior to the COVID-19 pandemic, and current influenza testing and treatment patterns might be affected by practices, behaviors, etc. related to the pandemic. Due to our limitations, it further highlights the importance for continuing investigation of differences over time to think through meaningful influenza interventions aimed at metropolitan versus micropolitan.

## Conclusion

Overall, our analysis highlights differences in influenza testing and antiviral treatment in micropolitan versus metropolitan areas in the U.S. from 2010 to 2016. We found evidence of both lower testing rates and higher treatment rates in metropolitan versus micropolitan areas. These discrepant results highlight the difficulty in understanding variation in influenza burden by rurality in the United States based on any one indicator in isolation. Further, the results lead to questions about potential disparities in influenza testing and treatment in micropolitan areas of the United States which could be driven by a variety of access and sociobehavioral factors. Our findings highlight that further research into the complex relationship of rurality and health disparities for influenza would be beneficial. Public health response to the burden of influenza and issues of resource allocation arise every year during the winter influenza season in the United States, and may be even more critical during a future influenza pandemic. Identifying patterns and differences of influenza across a variety of indicators helps to identify where disparities exist and may need targeted efforts to improve the prevention and control of influenza.

## Electronic supplementary material

Below is the link to the electronic supplementary material.

Supplementary Material 1

## Data Availability

The aggregate data that support the findings of this study are available on reasonable request from the corresponding author and must be approved by FDA and CDC working partners. The data are not publicly available.

## Abbreviations

AV: Antiviral
RIDT: Rapid influenza diagnostic test
CBSA: Core based statistical area
MSA: Metropolitan statistical area
MuSA: Micropolitan statistical area
CMS: Centers for Medicare and Medicaid Services
PDP: Prescription Drug Plans
CDC: Centers for Disease Control and Prevention
ILINET: Influenza-like illness Surveillance Network
ILI: Influenza-like illness
CAR: Conditional autoregressive
WAIC: Widely Applicable Information Criterion
RR: Rate ratio
CI: Confidence interval
