# Supplementary material for: Differences in influenza testing and treatment in micropolitan versus metropolitan areas in the U.S. using medicare claims data from 2010 to 2016
Source: BMC Public Health. 2025 Jan 23;25:291. doi: 10.1186/s12889-025-21555-4 (PMC11761195; doi:10.1186/s12889-025-21555-4)
Supplement: Supplementary file 1 — Supplementary Material 1 [file 12889_2025_21555_MOESM1_ESM.docx]

Supplemental

Supplemental Image 1: Map of core-based statistical areas by micropolitan statistical areas (red) and metropolitan statistical areas (blue).


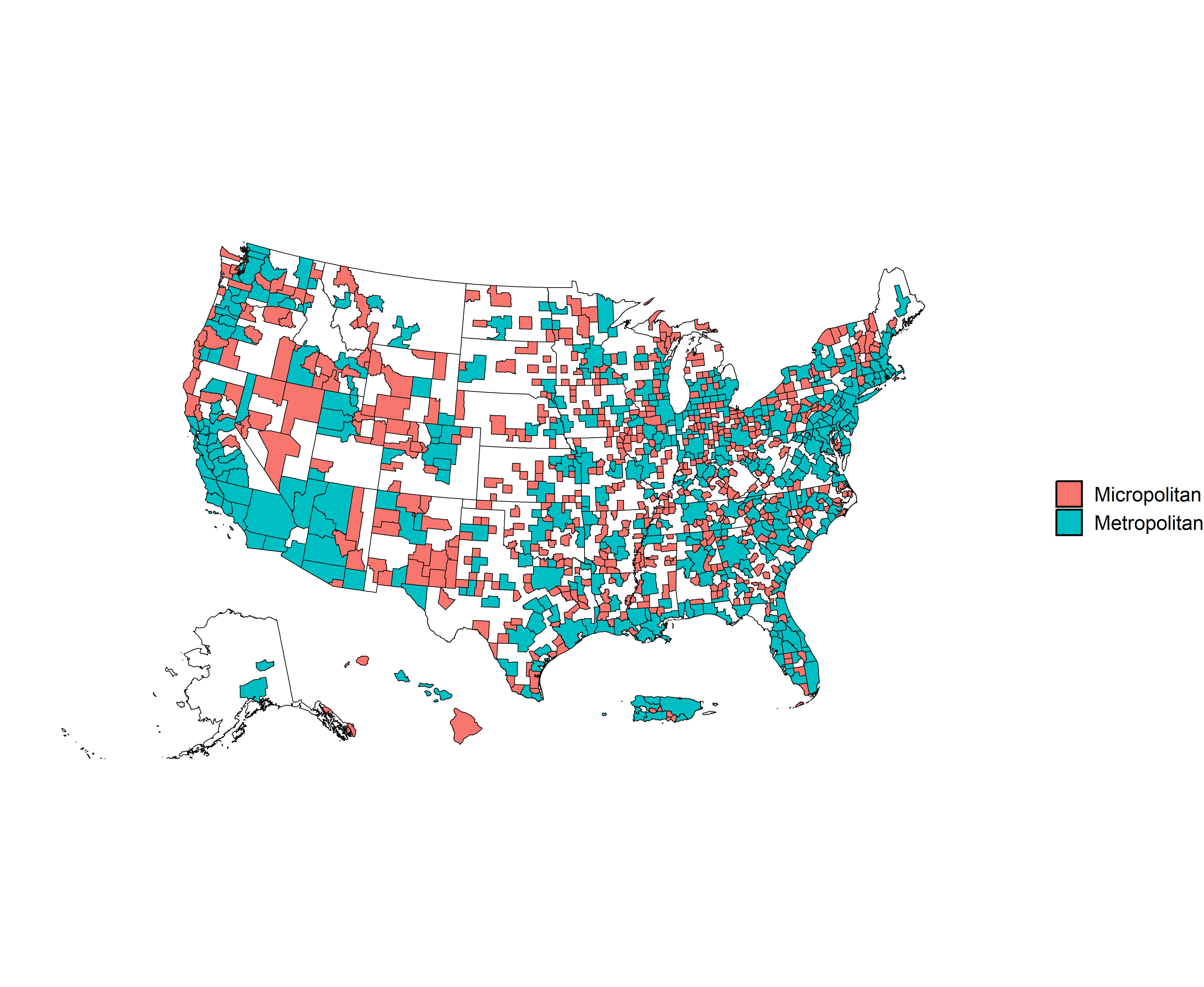


Supplemental Table 1: Coefficients from multivariate models for rapid influenza diagnostic tests (RIDT) model and antiviral (AV) model for all weeks and for only high influenza activity weeks.

| **Variables** | **RIDT**  **Exp(Coefficient) (95% CI)** | | **AV**  **Exp(Coefficient) (95% CI)** | |
| --- | --- | --- | --- | --- |
|  | **All Weeks** | **High Only** | **All Weeks** | **High Only** |
| Intercept | 5.0x10^-5^ (4.7x10^-5^ – 5.4x10^-5^) | 4.1x10^-4^ (3.8x10^-4^ – 4.4x10^-4^) | 2.5x10^-5^ (2.4x10^-5^ – 2.7x10^-5^) | 3.6x10^-4^ (3.4x10^-4^ – 3.8x10^-4^) |
| MSA | 0.84 (0.73 – 0.94) | 0.82 (0.73– 0.92) | 1.12 (1.04 – 1.22) | 1.15 (1.06 – 1.24) |
| Population Density | 0.89 (0.84 – 0.94) | 0.85 (0.81 – 0.91) | 0.85 (0.82 – 0.89) | 0.86 (0.82 – 0.89) |
| Temperature | 0.98 (0.96 – 0.99) | 0.95 (0.92 – 0.99) | 1.02 (1.00 – 1.04) | 0.87 (0.83 – 0.92) |
| Humidity | 1.01 (0.99 – 1.02) | 1.04 (0.99 – 1.09) | 0.92 (0.90 – 0.94) | 1.10 (1.03 – 1.16) |
| Under Poverty Line | 0.96 (0.90 – 1.03) | 0.95 (0.92– 0.99) | 1.09 (1.05 – 1.14) | 1.10 (1.05 – 1.14) |
| Over 85 years | 0.91 (0.85 – 0.97) | 0.91 (0.85 – 0.96) | 0.98 (0.94 – 1.02) | 0.99 (0.95 – 1.03) |
| Unemployed | 0.98 (0.88 – 1.02) | 0.93 (0.87 – 1.00) | 1.05 (1.00 – 1.11) | 1.02 (0.97 – 1.07) |
| High School Education | 0.89 (0.82 – 0.96) | 0.91 (0.85 – 0.98) | 1.07 (1.01 – 1.12) | 1.07 (1.02 – 1.12) |
| Medicare Only | 1.15 (1.09 – 1.23) | 1.14 (1.08 – 1.20) | 1.18 (1.14 – 1.23) | 1.16 (1.12 – 1.21) |
| Home Owners | 1.02 (0.94 – 1.11) | 1.00 (0.93 – 1.07) | 1.11 (1.06 – 1.17) | 1.10 (1.05 – 1.15) |
| No Vehicle | 0.90 (0.85 – 0.96) | 0.90 (0.85 – 0.94) | 1.00 (0.96 – 1.04) | 0.97 (0.94 – 1.01) |
| 60+ Minute Commute | 1.02 (0.95 – 1.08) | 1.01 (0.95 – 1.07) | 1.03 (0.98 – 1.07) | 1.01 (0.97 – 1.05) |
| Median House Value | 0.72 (0.67 – 0.78) | 0.74 (0.69 – 0.79) | 1.02 (0.98 – 1.07) | 1.00 (0.96 – 1.04) |
| Non-White | 1.14 (1.05 – 1.23) | 0.98 (0.91 – 1.05) | 1.46 (1.38 – 1.53) | 1.19 (1.13 – 1.25) |
| Season 11/12 | 1.05 (1.03 – 1.07) | 0.58 (0.64 – 0.63) | 0.86 (0.83 – 0.88) | 0.45 (0.41 – 0.50) |
| Season 12/13 | 1.87 (1.83 – 1.90) | 1.96 (1.90 – 2.02) | 1.88 (1.83 – 1.93) | 2.25 (2.16 – 2.35) |
| Season 13/14 | 2.02 (1.98 – 2.06) | 1.70 (1.64 – 1.77) | 1.71 (1.67 – 1.76) | 1.39 (1.32 – 1.46) |
| Season 14/15 | 3.21 (3.16 – 3.27) | 3.62 (3.51 – 3.73) | 2.73 (2.66 – 2.80) | 3.94 (3.78 – 4.11) |
| Season 15/16 | 2.90 (2.85 – 2.96) | 1.62 (1.54 – 1.70) | 1.72 (1.67 – 1.77) | 1.32 (1.23 – 1.41) |
| cos (2πt/52.25) | 0.50 (0.49 – 0.51) |  | 0.39 (0.38 – 0.40) |  |
| sin (2πt/52.25) | 4.45 (4.34 – 4.57) |  | 5.50 (5.31 – 5.71) |  |
| cos (4πt/52.25) | 1.33 (1.31 – 1.36) |  | 1.21 (1.28 – 1.35) |  |
| sin (4πt/52.25) | 0.75 (0.73 – 0.76) |  | 0.60 (0.58 – 0.61) |  |
| cos (2πt/52.25)* Season 11/12 | 1.21 (1.12 – 1.24) |  | 1.15 (1.10 – 1.19) |  |
| cos (2πt/52.25)* Season 12/13 | 1.08 (1.06 – 1.11) |  | 1.17 (1.13 – 1.21) |  |
| cos (2πt/52.25)* Season 13/14 | 1.21 (1.18 – 1.21) |  | 1.20 (1.17 – 1.25) |  |
| cos (2πt/52.25)* Season 14/15 | 0.98 (0.96 – 1.00) |  | 0.97 (0.94 – 1.00) |  |
| cos (2πt/52.25)* Season 15/16 | 0.95 (0.93 – 0.98) |  | 0.91 (0.88 – 0.95) |  |
| sin (2πt/52.25)* Season 11/12 | 0.51 (0.50 – 0.53) |  | 0.40 (0.38 – 0.41) |  |
| sin (2πt/52.25)* Season 12/13 | 1.27 (1.27 – 1.30) |  | 1.61 (1.54 – 1.67) |  |
| sin (2πt/52.25)* Season 13/14 | 0.81 (0.79 – 0.83) |  | 0.75 (0.72 – 0.78) |  |
| sin (2πt/52.25)* Season 14/15 | 1.26 (1.22 – 1.29) |  | 1.82 (1.75 – 1.90) |  |
| sin (2πt/52.25)* Season 15/16 | 0.59 (0.58 – 0.61) |  | 0.51 (0.49 – 0.53) |  |
| cos (4πt/52.25)* Season 11/12 | 1.09 (1.06 – 1.11) |  | 1.32 (1.27 – 1.37) |  |
| cos (4πt/52.25)* Season 12/13 | 0.63 (0.61 – 0.64) |  | 0.46 (0.45 – 0.48) |  |
| cos (4πt/52.25)* Season 13/14 | 0.65 (0.63 – 0.66) |  | 0.54 (0.52 – 0.56) |  |
| cos (4πt/52.25)* Season 14/15 | 0.61 (0.59 – 0.62) |  | 0.44 (0.42 – 0.45) |  |
| cos (4πt/52.25)* Season 15/16 | 1.20 (1.18 – 1.23) |  | 1.48 (1.43 – 1.53) |  |
| sin (4πt/52.25)* Season 11/12 | 1.35 (1.32 – 1.39) |  | 1.61 (1.56 – 1.67) |  |
| sin (4πt/52.25)* Season 12/13 | 1.21 (1.19 – 1.24) |  | 1.31 (1.27 – 1.35) |  |
| sin (4πt/52.25)* Season 13/14 | 1.35 (1.32 – 1.38) |  | 1.58 (1.53 – 1.63) |  |
| sin (4πt/52.25)* Season 14/15 | 1.34 (1.31 – 1.36) |  | 1.57 (1.52 – 1.63) |  |
| sin (4πt/52.25)* Season 15/16 | 1.53 (1.49 – 1.56) |  | 1.50 (1.53 – 1.65) |  |
